# Positive Residential Care Integration Scale: Portuguese Adaptation and Validation

**DOI:** 10.3390/ejihpe15120252

**Published:** 2025-12-09

**Authors:** Ana Simão, Cátia Martins, Elias Ratinho, Brianne H. Kothari, Cristina Nunes

**Affiliations:** 1 University Research Center in Psychology (CUIP), Universidade do Algarve, Campus de Gambelas, 8005-135 Faro, Portugal; jeratinho@ualg.pt (E.R.); csnunes@ualg.pt (C.N.); 2 Oregon State University-Cascades, 650 SW Columbia, Bend, OR 97702, USA; brianne.kothari@osucascades.edu

**Keywords:** adolescents, positive home integration, psychometric properties, residential care

## Abstract

Young people in residential care settings hold distinct preferences regarding their relationships with key adults, including caseworkers and caregivers. However, their perspectives are not consistently assessed or effectively integrated into case planning. Evaluating this integration is essential for fostering positive adjustment and placement stability. Given that residential care represents the predominant child welfare intervention in Portugal, this study adapts and validates an existing instrument for use with youth in residential care institutions, providing evidence of its validity and reliability. Self-report questionnaires—the Positive Residential Care Integration (PRCI) scale (an adapted Positive Home Integration scale) and Strengths and Difficulties Questionnaire—were administered to 511 youth (279 girls and 232 boys), aged 12 to 24 years, across 46 Portuguese residential care institutions. The study examined the face validity, discriminant validity, and reliability of the PRCI scale. Confirmatory factor analysis indicated good model fit, supporting a unidimensional six-item structure. Correlation analyses demonstrated associations with psychological adjustment and sociodemographic variables. The PRCI scale showed satisfactory psychometric properties, confirming its reliability for assessing youth integration in residential care. Cross-gender measurement invariance was also confirmed. These findings underscore the instrument’s relevance and validity for evaluating integration within residential settings and provide valuable guidance for caregivers, professionals, and caseworkers in child welfare services.

## 1. Introduction

Residential care is a protective provision established to uphold the rights and welfare of children and young people at risk, as outlined in the Portuguese Law for the Protection of Children and Young People in Danger (Law n.º 142/2015, 2015). These institutions are designed to support young people’s life plans, safeguard their protection, and uphold their rights through individualized interventions tailored to each individual’s specific needs, continuing until they reach the age of 25. This includes the provision of age-appropriate care tailored to their personal characteristics.

Residential care settings (RCS) are required to guarantee the highest possible well-being, equal opportunities, and the fulfillment of specific developmental needs. They also support the acquisition of autonomy skills and the effective promotion and exercise of young people’s rights, without discrimination based on personal characteristics (e.g., age, religion). Currently, there are no standardized methods for evaluating these parameters, and each institution maintains autonomy in carrying out these responsibilities. Institutions employ permanent teams of qualified professionals (Ordinance n.º 450/2023, 2023) who provide specialized support in children’s daily lives.

Despite existing legislation and protection services, young people in RCS are at heightened risk of experiencing emotional, behavioral, and social difficulties compared to peers in alternative care arrangements (Assouline & Attar-Schwartz, 2020; Simkiss et al., 2013). In Portugal, by the end of 2023, more than 6400 youth were under the care of the welfare system, with 84% placed in residential care institutions. Approximately 70% of these youth were between 12 and 20 years of age (Instituto da Segurança Social, 2024).

From an ecosystemic perspective—such as the conceptual framework proposed by James and Meezan (2002)—examining children in out-of-home (OoH) placements requires consideration of relationships across several ecological levels. This begins with the child’s characteristics in interaction with features of their environment, such as adults, other youth, and the systems responsible for their care. This perspective enables a deeper understanding of outcomes related to changes in health as well as youth’s psychological, behavioral, and emotional functioning (Aguilar-Vafaie et al., 2011).

### 1.1. Relationship with Institutional Caregivers and Other Youth

Research has underscored the importance of a consistent and nurturing caregiving environment for healthy development, as it supports the formation of secure and supportive relationships with caregivers (Attar-Schwartz, 2014). Changes in caregiving placement (e.g., institutionalization) entail not only a shift in environment but also a disruption of established caregiving relationships, which may negatively affect developmental outcomes (Almas et al., 2020). This disruption occurs because new caregiving relationships often lack the continuous emotional bonds typically formed with biological families (Cahill et al., 2016).

Staff in RCS play a pivotal role in fostering positive environments for youth by cultivating strong, supportive relationships. They are responsible for approximately 95% of the time young people spend in residential care, making their influence on developmental outcomes particularly significant (Anglin, 2019; Assouline & Attar-Schwartz, 2020; Cahill et al., 2016).

In Portuguese residential care institutions, multidisciplinary teams typically include a director, case managers—most often social workers, social educators, and psychologists—and residential caregivers (Carvalho, 2013; Instituto da Segurança Social, 2007). The director oversees service coordination, including the planning, implementing, and evaluation of annual intervention plans; management of financial resources; assurance of child protection and care quality; and attention to the needs of both youth and staff. The director also fosters collaboration with local authorities, community organizations, and other child-related professionals (Carvalho, 2013).

Case managers are responsible for assessing each youth’s needs, identifying appropriate services, advocating on their behalf, and developing individualized intervention plans in close cooperation with child protection agencies (Carvalho, 2013). Residential caregivers, who generally work under the supervision of case managers, form the frontline staff providing daily support to young people in care (Jordan et al., 2009). Their responsibilities include organizing and maintaining daily routines within the RCS and offering socioemotional and educational support, such as assisting with bedtime and morning routines or helping with schoolwork (Bastiaanssen et al., 2012).

The role of caregivers encompasses both parental and social functions. Parental tasks include feeding, hygiene, household management, discipline, counseling, and the teaching of life skills, while social functions involve engaging with youth through play, conversation, and companionship. Providing emotional support, maintaining availability, and responding appropriately to the specific needs of young people are central aspects of caregiving. Relationships between caregivers and youth are often characterized by intimacy, trust, warmth, and supportive practices (Bastiaanssen et al., 2012; Cahill et al., 2016). Meanwhile, technical team members monitor the progress of each youth, assess their circumstances, and define and implement their protection plan in accordance with court decisions or directives from child and youth protection commission (Law n.º 142/2015, 2015; Ordinance n.º 450/2023, 2023).

Some of the most meaningful experiences for young residents occur through daily interactions, where caregivers play a crucial role in fostering consistent and positive connections. Such interactions promote a sense of security and well-being and support the development of alternative, secure attachment figures (Assouline & Attar-Schwartz, 2020; Harder et al., 2013). Establishing supportive relationships with caregivers is therefore vital for young people in RCS (Cahill et al., 2016). However, high caregiver turnover rates and unfavorable staff-to-youth ratios can hinder this process. Consequently, RCS should strive to cultivate a positive and predictable environment while promoting opportunities for youth to build networks of trusting social relationships (Anglin, 2019).

Social support—particularly from adults in extrafamilial contexts (e.g., teachers, institutional staff)—plays a critical role in academic performance, social and personal adaptation, and psychological adjustment (Assouline & Attar-Schwartz, 2020). The value youth place on this support serves as an indicator of institutional quality (Attar-Schwartz, 2011; Martín, 2011). Therefore, caregivers must provide the emotional support and affection that these young people require, as research indicates that forming strong attachments to significant adults act as a protective factor during the transition out of the welfare system (Alves et al., 2024; Martín, 2011; Martín & Dávila, 2008).

### 1.2. Residential Care Institutions

According to the Report on the Transition from Institutional Care to Community-Based Services in 27 EU Member States (Šiška & Beadle-Brown, 2020), institutionalization remains the predominant form of care provision for children and youth without parental care or in psychosocial danger across several European Union countries. In Portugal, there continues to be an overreliance on institutional care for young people in alternative care arrangements.

Although several instruments have been developed to assess contextual, environmental, and social characteristics of youth in OoH placements—such as the Emotional Climate in Residential Care Scale for Youth (Santos et al., 2023), Foster Home Integration (Leathers, 2006), Place Attachment Scale (Magalhães & Calheiros, 2015b), Rights Perception Scale (Magalhães, 2015, as cited in Magalhães & Calheiros, 2020), Group Climate Instrument for Children (Strijbosch et al., 2014), Group Identification Scale (Magalhães & Calheiros, 2015a), and Revised Social Climate Scale (Colton, 1989)—there remains a need to develop short, self-report measures specifically designed for youth residing in RCS. Such instruments should capture young people’s perspectives on their integration and their perceptions of how effectively their needs are understood and met by various stakeholders involved in their care. The brevity of these instruments is particularly important. Short and focused measures can reduce completion time and minimize missing data resulting from fatigue or lack of motivation, which are known to increase refusal rates (Stanton et al., 2002).

A report from the Eurochild network (UNICEF & Eurochild, 2021) highlights the complete absence of Portuguese youth’s perspectives on the care they receive within existing data on young people in OoH settings. Growing attention in the field of residential care has been directed toward the concept of “child participation”, following Portugal’s ratification of the United Nations Convention on the Rights of the Child (United Nations, 1989), particularly Article 12, which guarantees children the right to express their views on matters affecting them and to have those views duly considered. Child participation refers to the meaningful involvement of children in decisions that influence their lives. Research indicates that limited opportunities for participation are associated with poorer mental health outcomes (Engel de Abreu et al., 2023).

### 1.3. Measuring Youth Integration in OoH Placements

Several constructs in the literature relate to the integration of young people in OoH placements, and various instruments have been developed to measure them. However, given the diversity of care settings, it is important to recognize that, within RCS, the relationship between youth and caregivers plays a pivotal role in fostering positive adjustment, placement stability, and reducing placement disruptions (Leathers, 2006; Rauktis et al., 2011).

Place attachment is commonly defined as a multidimensional construct that describes the emotional bond between individuals and specific places (Friesinger et al., 2022; Lewicka, 2011). It encompasses symbolic, emotional, and functional connections with a place that involve both physical and social dimensions (Raymond et al., 2010; Williams & Vaske, 2003) as well as underlying psychological processes, such as feelings of affection and belonging (Scannell & Gifford, 2010). These connections may be grounded in social or environmental attributes, as well as in personal meanings and self-reflective experiences.

Place attachment has been positively associated with individual well-being (Junot et al., 2018; Rollero & De Piccoli, 2010), quality of life (Azevedo et al., 2013; Friesinger et al., 2022), and positive social relationships. Both social and material dimensions can be understood as predictors of the emotional bonds formed by people with places (Friesinger et al., 2022). In this regard, a positive bond with a place can help self-regulation processes for managing negative emotions (Scannell & Gifford, 2010). It is therefore essential to prioritize well-being alongside, rather than solely focusing on psychopathology, and to examine the significance of individual’s relationships with their living environments, as these factors are critical for overall adjustment and mental health (Magalhães & Calheiros, 2020). Research further indicates that young people’s perceptions of their rights within the welfare system predict lower levels of maladjustment (Magalhães et al., 2016).

The quality of RCS depends on their capacity to provide youth with opportunities for participation in decision-making processes, respect for their privacy and identity, and the promotion of autonomy (Del Valle et al., 2012). Accordingly, it can be hypothesized that young people’s relationship with residential facilities and their integration into the institutional context play a crucial role in their well-being and adjustment. These youth have often experienced the loss of their family home and multiple relocations within the child welfare system, making attachment to place and relationships with people particularly important.

Existing measures assessing youth’s experiences of home integration are generally lengthy and primarily focused on foster home placements and foster families, often emphasizing the perspectives of caseworkers or caregivers rather than those of the youth themselves. Various approaches have been used to operationalize constructs relevant to this population, including foster home adjustment and adaptation (Affronti et al., 2015; Jones et al., 2016), foster home integration (Leathers, 2006), child-caregiver alliance (Rauktis et al., 2011), relational permanence (The Youth Connections Scale, Jones & LaLiberte, 2013), and other dimensions of child welfare practice such as child behavior (Leathers, 2002) and placement stability (Leathers, 2006; Waid et al., 2016). However, many studies have not tested measurement invariance by sex due to limited sample sizes (Santos et al., 2023; Waid et al., 2017).

To the best of our knowledge, only one empirically validated, youth-reported measure has been developed to assess young people’s integration into foster homes: the Positive Home Integration (PHI) scale (Kothari et al., 2018). The PHI is a nine-item instrument that asks youth to evaluate the quality of their relationships with their primary foster caregiver and the degree to which they feel integrated into the foster family. It captures young people’s sense of belonging and their perceptions of relationships with caregivers, considering the impact of integration on mental health and well-being, as well as the importance of feeling understood, supported, and having their needs met (Kothari et al., 2018).

In validation studies with American youth, PHI scores were significantly correlated with established youth-reported measures of well-being and caregiver-reported assessments (Waid et al., 2017). The findings demonstrated that preadolescent and adolescent youth can reliably report their perceptions of integration within foster homes using a brief self-report instrument. Thus, the PHI provides a concise, psychometrically sound measure for capturing multiple dimensions of the foster care experience from the youth’s perspective (Kothari et al., 2018).

The PHI items (e.g., To what extent/how much do you feel included in your (foster) family?; How well do you get along with your (foster) parent?) are rated on a 10-point Likert scale, with higher scores indicating stronger relationships with caregivers and a greater sense of integration into the foster household (Waid et al., 2017). The scale demonstrated strong internal consistency (Cronbach’s α = 0.82–0.91) and satisfactory test–retest reliability, supporting its robustness. The PHI is therefore a useful tool for practitioners and researchers seeking to assess and promote the relational well-being of youth in foster care, as well as for professionals working within child welfare systems (Kothari et al., 2018).

#### Current Study

To date, and to the best of our knowledge, no measure has been adapted for the Portuguese population that succinctly captures the perspectives and experiences of youth in residential care, particularly regarding their relationships with caregivers and other significant adults across critical life domains (e.g., feeling respected, being heard). Moreover, there remains a need for a brief, psychometrically robust instrument that centers on youth’s perspectives of integration within RCS—defined as their sense of belonging, perceptions of relationships with professionals, and views on how their needs are addressed. This focus is especially important given the distinct roles that adults occupy within institutional environment.

The present study aimed to adapt and validate the Positive Home Integration scale (PHI; Kothari et al., 2018) for use in the Portuguese residential care context and to examine its dimensionality, reliability, and discriminant validity using external variables. This research is particularly relevant as it provides both scholars and practitioners with a reliable tool to assess young people’s perceptions of their experiences in residential care institutions.

It was hypothesized that the adapted version—the Positive Residential Care Integration (PRCI) scale—would demonstrate a unidimensional structure, strong internal consistency, discriminant validity, measurement invariance across gender, and significant associations with indicators of psychological adjustment among youth in RCS. Specifically, higher PRCI scores were expected among youth reporting positive relationships with caregivers and lower levels of internalizing and externalizing symptoms, whereas lower PRCI scores (typically 1–4 on a 10-point scale) were anticipated to correspond with poorer mental health outcomes.

## 2. Materials and Methods

### 2.1. Participants

Participants were 511 youth from 46 Portuguese residential care institutions, including 232 boys (45.4%) and 279 girls, aged between 12 and 24 years (*M* = 15.87, *SD* = 2.25). The age distribution was as follows: 12–15 years (*n* = 226, 44.2%), 16–18 years (*n* = 227, 44.4%), and 19–24 years (*n* = 58, 11.4%). The 12–24 age range is the most prevalent among young people in Portuguese residential care (Instituto da Segurança Social, 2024), and prior studies have also included individuals over 18 years of age (e.g., Alves et al., 2024). The inclusion of participants aged 18–24 years was justified by their cohabitation with younger residents within the same institutions—often sharing bedrooms and facilities—and their adherence to identical institutional rules and routines regardless of age (e.g., completing the same tasks to receive a weekly allowance, observing the same curfew for returning to the institution, and surrendering mobile phones at bedtime, even when needed for schoolwork). The interaction between young people over 18 and younger residents often occurs while they await placement in units for support and promotion of autonomy.

On average, participants had been living in their current institution for 41 months (*SD* = 43; range = 1–204). Although many youth presented multiple reasons for placements, the most frequently reported grounds for protection measures were neglect and abuse (38%), school absenteeism (30%), financial hardship (29%), and domestic violence (25%). Regarding education, most participants (75%) were enrolled in middle school. The majority (93%) had siblings, but only 31% were residing in the same institution.

### 2.2. Measures

#### 2.2.1. Positive Residential Care Integration (PRCI) Scale

The PRCI scale is an adaptation of the PHI scale (Kothari et al., 2018), a self-report, nine-item Likert-type instrument that assesses youths’ perceptions of inclusion within the foster home and the quality of their relationship with the primary foster caregiver.

Permission to translate and validate a Portuguese version of the PHI was obtained from the original authors. The standard back-translation procedure was followed (van de Vijver, 2016). The initial translation from English to Portuguese was conducted by the first and last authors, with careful attention to linguistic and contextual adaptation. Specifically, wording was modified so that youth could report on caregivers rather than parents (e.g., How well does institution’s technicians respond to your needs?) and refer to the institution instead of a foster home or family (e.g., To what extent/how much do you feel included in the institution?). These adaptations ensured that the items were contextually appropriate and easily understood by youth living in RCS.

Throughout the translation process, recommended cross-cultural adaptation procedures were followed to avoid item bias and differential item functioning (Hambleton et al., 2004). Discrepancies between translators were resolved through discussion until full semantic equivalence between the English and Portuguese versions was achieved. The translated PRCI was then pilot-tested with a small group of youths, of varying ages and educational backgrounds, who were not included in the main sample. This pilot testing confirmed that the items were comprehensible, age-appropriate, and clearly referred to the intended adults (caregivers or staff).

The results section presents analyses of the PRCI’s psychometric properties, including dimensionality, reliability, validity, and measurement invariance.

#### 2.2.2. Strengths and Difficulties Questionnaire (SDQ, R. Goodman, 1997; Portuguese Version by Pechorro et al., 2011)

The SDQ is a 25-item self-report instrument designed to assess socioemotional problems. Items are rated on a three-point Likert-type scale (0 = “Not true”, 1 = “Somewhat true”, 2 = “Certainly true”) and are grouped into five subscales: emotional problems, behavioral problems, hyperactivity/inattention difficulties, peer relationship problems, and prosocial behaviors (R. Goodman, 1997). The total difficulties score—calculated by summing all subscales except prosocial behaviors—serves as a psychometrically supported indicator of overall child mental health problems (A. Goodman et al., 2010). In the original version (R. Goodman, 2001) Cronbach’s alpha coefficients ranged from 0.41 to 0.80, indicating acceptable to good internal reliability across most subscales.

In the Portuguese adaptation by Pechorro et al. (2011), Cronbach’s alphas ranged from 0.43 to 0.61. In the present study, reliability coefficients for the SDQ were as follows: emotional problems α = 0.68, behavioral problems α = 0.57, hyperactivity/inattention difficulties α = 0.66, peer relationship problems α = 0.51, prosocial behaviors α = 0.78, and total scale α = 0.78.

Although some subscale alphas were below the conventional 0.70 threshold (Hair et al., 2019; Kline, 2023), this finding is not unexpected given that each subscale comprises only five items, which can lead to underestimation of internal consistency. To complement Cronbach’s alpha, average inter-item correlations were computed, as recommended by Clark and Watson (1995). These ranged from 0.18 to 0.41 across the five subscales (emotional problems = 0.30, behavioral problems = 0.22, hyperactivity/inattention difficulties = 0.27, peer relationship problems = 0.18, prosocial behaviors = 0.41). Most subscales demonstrated moderate inter-item correlations (≥0.30), indicating satisfactory internal consistency, though some fell slightly below this threshold.

Overall, despite modest alpha coefficients, the SDQ subscales demonstrated adequate internal consistency for research purposes.

### 2.3. Procedures

Ethical approval for this study was obtained from the Ethics Committee of the University of Algarve (CEUAlg No. 110/2023). All procedures were conducted in accordance with the ethical standards of the 1964 Helsinki Declaration and its subsequent amendments, as well as comparable ethical standards. The authors of the original PHI scale granted permission to use the measure with youth in RCS and authorized its adaptation and validation for the Portuguese context.

Using a convenience sampling method, 46 residential care institutions across mainland Portugal and the Azores and Madeira archipelagos were contacted by telephone and email. Directors of each institution were informed about the study’s aims and procedures, and all consented to participate. Data were collected in person by the first author in 16 institutions. For institutions located in geographically distant regions, questionnaires were mailed, and a designated professional from each institutions supervised data collection. Detailed written instructions and clarifications were provided by email when authorization was obtained and reiterated in the mailed materials. The first author remained available to respond to any questions throughout the process.

Inclusion criteria required participants to be at least 12 years old and proficient in Portuguese. Youths identified by institutional staff as having cognitive impairments were excluded. Eligible participants were informed about the study’s objectives and invited to participate voluntarily. They were assured that their participation was confidential and that they could withdraw at any time without consequence. Participants aged 16 years or older provided written informed consent, while consent for those under 16 was obtained from their legal guardians or the institution’s technical directors.

Participants completed an anonymous, structured self-report questionnaire within their residential care institutions. Questionnaires were administered individually or in small groups, depending on institutional preferences and participants’ availability. Data collection occurred between October 2023 and October 2024 and included participants from all regions of Portugal.

#### Data Analysis

Data analyses were performed using IBM SPSS 29.0 (IBM Corp., 2020) and JAMOVI (Gallucci & Jentschke, 2021). A Confirmatory Factor Analysis (CFA) was conducted using Maximum Likelihood Estimation (Epskamp et al., 2022) to examine the factorial structure of the PRCI scale. Model fit was evaluated using several goodness-of-fit indices, including Comparative Fit Index (CFI), Tucker–Lewis Index (TLI), chi-square/degrees of freedom ratio (χ^2^/df), Akaike Information Criterion (AIC), Standardized Root Mean Square Residual (SRMR), and Root Mean Square Error of Approximation (RMSEA) (R Core Team, 2023; Rosseel, 2012).

Model fit was considered acceptable when CFI and TLI values exceeded 0.90, RMSEA values were ≤0.10, and χ^2^/df values ranged between 2 and 5 (Kline, 2023). The model with the smallest AIC value was selected as the best-fitting model (Rosseel et al., 2023). The CFA was performed on the original scale items using a categorical correlation matrix. Items with standardized factor loadings above 0.50 were retained, as this threshold indicates significant and meaningful loadings (Pituch & Stevens, 2016). No modification indexes were used to improve model fit. Item skewness (S) and kurtosis (K) were also inspected, with values greater than 3.0 for skewness and 8.0 for kurtosis considered serious violations of normality (Kline, 2023; Marôco, 2021).

Factorial invariance of the instrument was assessed through Structural Equation Modeling (SEM) using RStudio (version 4.5.0) and the *lavaan* (version 0.6.19; Rosseel, 2012) and *semTools* (version 0.5-7; Jorgensen et al., 2025) packages. Following the recommendations of Milfont and Fischer (2010) and Putnick and Bornstein (2016), the analysis was conducted in sequential steps, testing configural, metric, and scalar models.

The configural model evaluated whether the one-factor structure provided an adequate and equivalent fit across gender groups (female and male), allowing all parameters to vary freely. In the metric model, factor loadings were constrained to equality across groups to examine the equivalence of the relationships between the items and the latent factor. The scalar model imposed additional constraints on the intercepts of the indicators to test the comparability of latent means.

All models were estimated using the Maximum Likelihood method with robust sandwich-type standard errors and Yuan–Bentler scaled corrections to account for potential deviations from normality. Model fit was evaluated using multiple indices: χ^2^/df, CFI, TLI, RMSEA, and SRMR.

To determine invariance across successive models, the criteria proposed by Cheung and Rensvold (2002) and Chen (2007) were applied, considering that differences smaller than 0.010 in CFI and 0.015 in RMSEA indicate invariance. Latent means and variances were estimated using the female group as the reference (mean fixed to zero and variance fixed to one).

As the data approximated a normal distribution, Pearson correlations coefficients were computed to explore associations between study variables. Correlation magnitudes were classified as low (<0.20), moderate (0.20–0.50), or high (>0.50) (Marôco, 2021). Internal consistency was examined through corrected item-total correlations (>0.20), Cronbach’s alpha coefficients (>0.70), and mean inter-item correlations (0.15–0.50) (Clark & Watson, 1995; Marôco, 2021).

## 3. Results

### 3.1. Descriptive Statistics

Table 1 presents the descriptive statistics for the nine PRCI items, including means, standard deviations, skewness, and kurtosis. No significant deviations from normality were detected, as all skewness and kurtosis values fell within acceptable ranges (|*S*| < 3.0; |*K*| < 8.0). Overall, item means were relatively high, ranging from 6.65 to 8.09, indicating generally positive perceptions of integration and relationships with caregivers among youth in RCS.

### 3.2. Confirmatory Factor Analysis

To examine the psychometric properties of the PRCI, we first tested whether the presumed unidimensional structure of the original PHI scale could be replicated using CFA with Maximum Likelihood Estimation.

Goodness-of-fit indices were calculated for both the original nine-item model and the refined six-item model (Table 2). The results supported the adequacy of the proposed model, with the one-factor, six-item solution demonstrating the best fit to the data [χ^2^(9) = 58.6, χ^2^/df = 6.51, CFI = 0.97, TLI = 0.95, RMSEA = 0.10]. Although the chi-square value was significant—likely due to the large sample size (N = 511)—the high CFI and TLI values, and the acceptable RMSEA indicate that the model achieved satisfactory overall fit.

Items 5, 7, and 9 were removed based on low factor loadings (<0.40) and considerations of face validity. Their removal did not compromise the conceptual coherence of the scale.

Table 3 presents the standardized factor loadings for the one-factor model estimated using the ML-Robust method with the total sample. All items showed strong positive associations with the latent construct, wth loadings ranging from 0.61 to 0.87. Cronbach’s alpha coefficients indicated good internal consistency across the PRCI (α_range_ = 0.84–0.88). Corrected item-total correlations were moderate to high (CITC_range_ = 0.57–0.79), supporting the reliability and cohesion of the scale in assessing youth integration within residential care.

The internal consistency of the PRCI was examined using Cronbach’s alpha (α = 0.87) and McDonald’s omega (ω = 0.88), the latter providing a more robust estimate of reliability for latent constructs. Both coefficients indicated good internal consistency.

Consistent with the original PHI scale, the PRCI demonstrated a unifactorial structure, with all six items explaining more than 60% of the total variance in the construct. Each item’s communality exceeded 0.60, indicating that all items contributed substantially to the latent factor representing positive residential care integration.

**Figure 1:**
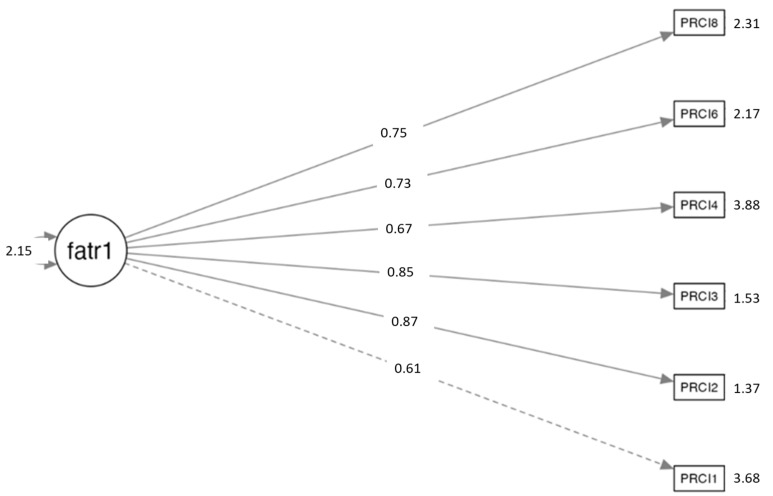
Path diagram of the confirmatory factor analysis.

Figure 1 displays the path diagram for the final one-factor, six-item solution obtained through CFA. It demonstrated that positive integration in residential care is associated with six items (Items 8, 6, 4, 3, 2, and 1) from the final solution. The numbers on the paths represent the standardized factor loadings, indicating strong relationships between each item and the latent factor. Item 1 exhibited the weakest loading, though it remained within acceptable limits. Error variances for each item are also presented, supporting the adequacy of the proposed measurement model.

### 3.3. Measurement Invariance Across Gender

The next step was to test for measurement invariance across gender (males vs. females). The multigroup analysis indicated a good overall fit for the configural model (CFI = 0.968, RMSEA = 0.105, SRMR = 0.030), demonstrating that the unidimensional structure of the PRCI scale is equivalent across sexes. Imposing metric invariance did not result in a significant deterioration of model fit (ΔCFI = 0.001, ΔRMSEA = −0.014, *p* = 0.79), and scalar invariance was also supported (ΔCFI = 0, ΔRMSEA = −0.008, *p* = 0.38). Thus, the PRCI scale demonstrated configural, metric, and scalar invariance between boys and girls, allowing for direct comparisons of latent means across groups. Finally, factor means were compared by fixing the female group mean to zero and estimating it in the male group. The latent mean difference was not statistically significant (*b* = 0.11, *SE* = 0.09, *p* = 0.23), indicating equivalent PRCI levels between boys and girls.

### 3.4. Discriminant Validity with External Variables and Scale Reliability

Discriminant validity was examined using correlations between the PRCI and the subscales of the SDQ, which assesses psychological adjustment through internalizing and externalizing dimensions.

As shown in Table 4, the PRCI was negatively correlated with behavior problems (*r* = −0.26, *p* ≤ 0.001), hyperactivity/inattention (*r* = −0.16, *p* ≤ 0.001), peer problems (*r* = −0.27, *p* ≤ 0.001), and the SDQ total difficulties score (*r* = −0.26, *p* ≤ 0.001). In contrast, a positive correlation was found with prosocial behavior (*r* = 0.38, *p* ≤ 0.001). These results provide evidence of discriminant validity, demonstrating that higher levels of perceived integration within residential care are associated with fewer socio-emotional and behavioral difficulties and greater prosocial functioning.

Convergent validity was supported by the significant correlations observed between the PRCI and SDQ subscales, particularly the positive association with prosocial behaviors and the negative association with the SDQ total difficulties score. The negative and significant correlation between the PRCI and total difficulties provides further evidence of construct validity, particularly given the acceptable reliability of the SDQ total scale.

Construct reliability was evaluated using Composite Reliability, which yielded a value of 0.89, indicating a high level of internal consistency among the PRCI items. To assess potential common method bias, Harman’s single-factor test using unrotated principal components analysis was conducted. The first factor accounted for 60% of the total variance, suggesting some influence of common method variance; however, this level does not indicate a serious threat to the validity of the findings.

Overall, these results reinforce the robustness and psychometric soundness of the PRCI as a measure of youth integration within RCS.

## 4. Discussion

Examining institutional integration provides critical insight into the factors shaping youth adjustment in RCS and can inform strategies to optimize their experiences following removal from biological families.

Previous research has highlighted that youth perceptions of their experiences in care and the quality of relationships with staff remain underexamined (Attar-Schwartz, 2014). Assessing these perceptions is particularly important for socially vulnerable and at-risk youth, as it offers key indicators of adjustment and well-being in a context they did not choose.

The primary aim of this study was to assess the psychometric properties of the PRCI scale among a Portuguese sample of 511 institutionalized youth. The PRCI evaluates youth perceptions of integration into RCS and their relationships with caregivers. Consistent with our hypothesis, the proposed unidimensional structure of the original PHI scale was replicated in this sample, demonstrating good reliability and discriminant validity with a measure of psychological adjustment.

CFA results supported a one-factor, six-item solution, with high factor loadings and acceptable fit indices. Three items were removed due to low factor loadings and limited face validity; these items primarily concerned institutional technicians rather than caregivers. This pattern likely reflects the greater frequency and quality of contact between youth and caregivers, who are often the closest and most influential adult figures to youth in RCS, providing daily socioemotional support, compared with technicians, whose roles are more administrative or coordination-focused (Anglin, 2019; Bastiaanssen et al., 2012; Cahill et al., 2016). Although directors are less involved in day-to-day interactions, they play a critical role in mediating conflicts and supporting the quality of youth-caregiver relationships (Magalhães et al., 2023). Since caregivers spend most of their time with youths, it is likely that integration is more influenced by relationships with them rather than by interactions with technicians who can be seen by young people as a link to the court, to child welfare processes, or to important decisions in their lives. The establishment of quality relationships positively impacts young people in RCS favoring emotional and behavioral functioning (Assouline & Attar-Schwartz, 2020; Magalhães & Calheiros, 2017; Magalhães et al., 2023).

As demonstrated by the Kothari et al. (2018) study, which validated a single-dimension measure with reliable and discriminant validity, our PRCI scale also showed good discriminant validity with measures of psychological adjustment. The PRCI demonstrated good internal consistency (α = 0.87, ω = 0.88), convergent validity as evidenced by negative correlations with SDQ total difficulties and subscales measuring behavioral and emotional problems, and positive correlations with prosocial behaviors. These findings align with prior research indicating that positive integration is associated with better psychological adjustment (Rodrigues et al., 2019; Soriano-Díaz et al., 2022).

PRCI showed strong measurement invariance across gender, indicating that observed scores are related to the latent construct. This suggests that the models do share some equivalence across these groups. It is important to mention that this is the first study testing for the measuring invariance of the PRCI scale in Portuguese youths.

Mean PRCI scores indicated generally high levels of perceived integration, consistent with findings from Kothari et al. (2018). Additionally, for the SDQ, the current work presented higher alpha coefficients than those reported in its validation study (Pechorro et al., 2011) across all subscales, with some subscales exceeding the results from R. Goodman’s (2001) study.

The bivariate correlations with various variables and measures demonstrated that the PRCI scale was associated with outcomes in the expected direction and showed significant associations with mental health outcomes (e.g., internalizing and externalizing problems), suggesting that improved institutional integration may be linked to better psychological adjustment.

Positive group climate in RCS—characterized by responsive professionals, opportunities for development, and a safe, structured environment—supports the formation of nurturing caregiver relationships and promotes youth well-being (Pinheiro et al., 2024; Strijbosch et al., 2014). Conversely, mental health difficulties can negatively influence social interactions and perceptions of group climate quality (Lanctôt et al., 2016; Leipoldt et al., 2019). Research indicates that placement quality significantly impacts youth adjustment during and after care, emphasizing the importance of assessing youth’s perceptions of their institutional environment (Hyde & Kammerer, 2009). The quality of relationships between youth and caregivers is a key predictor of successful adjustment and developmental outcomes in residential care (Assouline & Attar-Schwartz, 2020; Cahill et al., 2016; Harder et al., 2013; Silva et al., 2022).

Given that perceptions of an open and positive group climate predict intervention effectiveness and positive outcomes (Leipoldt et al., 2019), understanding the facilitators and barriers to a favorable residential environment can inform strategies to meet mental health needs and promote well-being, psychological adjustment, and satisfaction (Pinheiro et al., 2024). Positive integration in RCS can be characterized by mutual respect, kindness, feelings of inclusion, and opportunities to participate in decisions regarding their care process (Waid et al., 2017). Furthermore, successful integration appears to occur when individuals do not exhibit clinical-level emotional or behavioral symptoms, feel respected, can express their opinions, and have their needs adequately addressed (Assouline & Attar-Schwartz, 2020; Magalhães & Calheiros, 2017; Mota & Matos, 2015).

Previous research using self-report scales has shown that preadolescent and adolescent youth can reliably report their perceptions of integration within foster homes (Kothari et al., 2018) or group identification in RCS (Magalhães & Calheiros, 2015a). The current study confirms these findings using the PRCI scale, providing additional evidence of its validity and reliability. Following Kazdin (2021) framework, construct validity reflects the degree to which a measure captures the underlying concept it is intended to assess. Therefore, the PRCI appears to be a relevant and valid tool for evaluating youth perceptions of institutional integration. While correlations with some SDQ subscales should be interpreted cautiously, the primary contribution of this study is the provision of a brief, youth-centered assessment tool tailored to a Portuguese sample, offering new evidence supporting the validity and reliability of the construct’s dimensional structure. The Portuguese version of the six-item PRCI scale is provided in Appendix A.

### Limitations and Implications for Practice

The present study has several strengths, but some limitations should be acknowledged. First, data were based solely on youth self-reports of their integration into the institutional environment. While youth perspectives are crucial and underutilized in child welfare, incorporating caregiver reports could provide complementary insights. Second, social desirability bias may have influenced responses, as participants completed questionnaires within the residential care context, sometimes in the presence of directors, potentially inflating positive ratings. Third, the use of convenience sampling may have introduced selection bias, limiting the representativeness of the sample and the generalizability of findings to the broader population of institutionalized youth.

Additional psychometric limitations include the reliance on Harman’s single-factor test to examine common method bias; more rigorous methods are recommended for future research. Moreover, correlations with SDQ subscales with lower reliability should be interpreted cautiously. The cross-sectional design precludes examination of temporal stability or changes in integration over time, and test–retest reliability could not be assessed.

Future studies should employ additional criterion measures with established reliability to further validate the PRCI. Longitudinal designs are particularly important to track changes in youths’ integration, explore factors influencing these perceptions, and examine the development of relationships with caregivers over time. This is especially relevant given upcoming legal modifications in Portugal that are expected to alter institutional operations.

Despite these limitations, the study has notable strengths. It employed a national sampling frame, included youth perspectives on a critical developmental topic, and achieved high response rates in a hard-to-reach population. Although residential care contexts vary across countries, institutionalization remains a primary form of care for youth without parental care in many European countries, suggesting potential applicability of the PRCI beyond Portugal.

Importantly the PRCI demonstrates strong psychometric properties and provides a brief, youth-centered measure of integration, relationships with caregivers, and perceived participation in decision-making. This is particularly valuable as most youth enter care involuntarily. The PRCI can help prevent placement disruptions and promote stability by identifying youth with poorer integration. By allowing professionals to assess individual item responses, the scale facilitates targeted interventions, distinguishing youth who are thriving from those who require additional support. Overall, the PRCI offers a practical tool for both research and applied child welfare settings, supporting relationship-based interventions and promoting youth well-being in residential care contexts.

## 5. Conclusions

The existing literature highlights a lack of research on youths’ perceptions of their experiences in care and, consequently, the quality of their relationships with caregivers (Attar-Schwartz, 2014). The PRCI measure addresses this gap by enabling research focused on the integration of youth in RCS, a topic of growing importance over the past decade. Beyond its research value, the PRCI offers practical insights into youth perceptions of their living environments, supporting the evaluation of residential care approaches, the design of individualized interventions, and the professional development of practitioners working with this population.

The present study sought to adapt and validate a brief self-report measure for the Portuguese residential care context and to examine the factor structure of the PRCI among institutionalized youths. The findings demonstrated strong psychometric properties and invariance across gender, supporting the reliability and validity of the PRCI as a tool for use in child welfare and residential care contexts.

Overall, the results suggest that the PRCI can provide valuable information about how integration relates to youths’ psychological adjustment, experiences in care, and engagement with caregivers. By capturing young people’s own perspectives, this tool can help professionals identify individual needs, promote well-being, and foster a stronger sense of belonging within institutions.

In conclusion, the PRCI scale represents a brief, psychometrically sound, and youth-centered instrument with significant utility for both research and practice. It reinforces the notion that young people in residential care can reliably report on their relationships and integration experiences. Future studies should explore the PRCI’s predictive potential regarding youths’ overall adjustment, well-being, and long-term outcomes in care.

## Figures and Tables

**Table 1:** Descriptive Statistics of PRCI items.

PRCI Items	*M*	*SD*	*S*	*K*
Item 1: To what extent/how much do you feel included in the institution?	7.49	2.42	−0.85	0.06
Item 2: To what extent do you feel that you are treated with kindness in the institution?	7.42	2.40	−0.84	−0.03
Item 3: To what extent do you feel that you are treated with respect in the institution?	7.50	2.32	−0.79	−0.10
Item 4: To what extent do you feel that you are involved in decision making in the institution?	6.65	2.64	−0.47	−0.64
Item 5: On a scale of 1–10, how good is your relationship with institution’s technicians?	8.02	2.27	−1.22	0.79
Item 6: How well do you get along with the institution’s care workers?	8.09	2.14	−1.21	0.95
Item 7: On a scale of 1–10, how well does institution’s technicians listen to you?	7.73	2.30	−0.95	0.26
Item 8: How well does institution’s technicians respond to your needs?	7.74	2.29	−0.97	0.26
Item 9: When you have a problem, how well does institution’s technicians respond to you?	7.86	2.31	−1.07	0.41

*M* = Mean; *SD* = Standard deviation; *S* = Skewness; *K* = Kurtosis.

**Table 2:** Goodness of Fit Indices for tested Model of PRCI with CFA.

Models/Indices	χ^2^/df	CFI	TLI	RMSEA	RMSEA 90% CI	SRMR	AIC
1-Factor 9-items	401/27	0.89	0.85	0.17	0.15–0.18	0.06	18,014
1-Factor 6-items (without item 5, 7, and 9)	58.6/9	0.97	0.95	0.10	0.08–0.13	0.03	12,497

Note. χ^2^/df = Chi-square/degrees of freedom; CFI = Comparative Fit Index; TLI = Tucker–Lewis Fit Index; RMSEA = Root Mean Square Error of Approximation; CI = Confidence interval; SRMR = Standardized Root Mean Square Residual; AIC = Akaike Information Criterion.

**Table 3:** Items loadings, alpha if item deleted, and corrected item-total correlation.

Items	*β*	Alpha if Item Deleted	CITC
Item 01	0.61	0.88	0.57
Item 02	0.87	0.84	0.79
Item 03	0.85	0.85	0.77
Item 04	0.67	0.87	0.64
Item 06	0.73	0.86	0.68
Item 08	0.75	0.86	0.71

Note. *β* = Item Loadings; CITC = corrected item-total correlation.

**Table 4:** Descriptive statistics and correlations between PRCI and SDQ (N = 511).

	1	2	3	4	5	6	7
1. SDQ total score	-	0.74 ***	0.71 ***	0.72 ***	0.63 ***	−0.15 ***	−0.26 ***
2. Emotional problems		-	0.27 ***	0.38 ***	0.36 ***	0.22 ***	−0.07
3. Behavior problems			-	0.44 ***	0.33 ***	−0.31 ***	−0.26 ***
4. Hyperactivity/Inattention				-	0.15 ***	−0.15 ***	−0.16 ***
5. Peer problems					-	−0.21 ***	−0.27 ***
6. Prosocial behaviors						-	0.38 ***
7. PRCI							-
*M* (*SD*)	15.55	4.61	2.92	4.48	3.34	7.37	68.49
	(6.24)	(2.42)	(2.03)	(2.38)	(2.04)	(2.31)	(16.63)

*** *p* < 0.001.

## Data Availability

The data can be made available for consultation from the corresponding author upon request.

## Abbreviations

The following abbreviations are used in this manuscript:

PRCI	Positive Residential Care Integration
RCS	Residential Care Settings
SDQ	Strengths and Difficulties Questionnaire
OoH	Out-of-Home
PHI	Positive Home Integration
CFA	Confirmatory Factor Analysis

## Appendix A

**Table A1:** Portuguese version of the PRCI 6 items.

Items	
1	Quanto é que te sentes integrado/a na instituição?
2	Em que medida sentes que és tratado/a com gentileza na instituição?
3	Em que medida sentes que és tratado/a com respeito na instituição?
4	Em que medida sentes que na instituição te incluem nas decisões?
6	Como é a tua relação com os auxiliares da instituição?
8	Como achas que os técnicos da instituição respondem às tuas necessidades?
